# Prevalence of Multiple Enteroviruses Associated with Hand, Foot, and Mouth Disease in Shijiazhuang City, Hebei Province, China: Outbreaks of Coxsackieviruses A10 and B3

**DOI:** 10.1371/journal.pone.0084233

**Published:** 2014-01-02

**Authors:** Huifang Tian, Yong Zhang, Qiang Sun, Shuangli Zhu, Xiujuan Li, Zhuo Pan, Wenbo Xu, Baohong Xu

**Affiliations:** 1 Microbiology Laboratory, Shijiazhuang Center for Disease Control and Prevention, Shijiazhuang, Hebei, People’s Republic of China; 2 WHO WPRO Regional Polio Reference Laboratory, Ministry of Health Key Laboratory for Medical Virology, National Institute for Viral Disease Control and Prevention, Chinese Center for Disease Control and Prevention, Beijing, People’s Republic of China; Imperial College London, United Kingdom

## Abstract

Hand, foot, and mouth disease (HFMD) has been one of the most common infectious diseases in Shijiazhuang City, as is the situation in China overall. In the National HFMD surveillance system, the pathogen detection was focused on EV-A71 and CVA16, and therefore, information on the other EVs is very limited. In order to identify the circulating EV serotypes in the HFMD outbreaks in Shijiazhuang City during 2010–2012, 4045 patients presented with HFMD were recruited in the study, and clinical samples were investigated. Typing of EV serotypes was performed using the molecular typing methods, and phylogenetic analyses based on entire *VP1* sequences of human enterovirus 71 (EV-A71), coxsackievirus A16 (CVA16), CVA10 and CVB3 was performed. The results revealed that EV-A71 and CVA16 were the 2 most important pathogens but the circulating trends of the 2 viruses showed a shift, the spread of EV-A71 became increasingly weak, whereas the spread of CVA16 became increasingly stronger. CVA10 and CVB3 were the third and fourth most prevalent pathogens, respectively. Co-infection of two viruses at the same time was not found in these samples. Based on entire *VP1* region sequences, the phylogenetic analysis revealed that C4a subgenotype EV-A71, B1a and B1b subgenotype CVA16 continued to evolve. The CVA10 strains were assigned to 4 genotypes (A–D), whereas the CVB3 strains were assigned to 5 genotypes (A–E), with clear geographical and temporal-specific distributions. The Shijiazhuang CVA10 sequences belonged to 4 epidemic lineages within genotype C, whereas the Shijiazhuang CVB3 sequences belonged to 2 epidemic lineages within genotype E, which may have the same origins as the strains reported in other part of China. CVA10 and CVB3, 2 pathogens that were previously infrequently detected, were identified as pathogens causing the HFMD outbreaks. This study underscores the need for detailed laboratory-based surveillances of HFMD in mainland China.

## Introduction

Human enteroviruses (EVs) are members of the genus *Enterovirus* within the family *Picornaviridae*, order *Picornavirales*, which consists of 4 species: EV-A, EV-B, EV-C, and EV-D [1]. To date, EVs comprise more than 100 serotypes and are the most common pathogens that infect humans, especially children, worldwide. Most EV infections are asymptomatic or present with some benign symptoms, but they may also lead to serious illnesses such as acute flaccid paralysis, encephalitis, myocarditis, and encephalomyelitis [2], [3].

Hand, foot, and mouth disease (HFMD) is a common infectious disease of young children, particularly those less than 5 years of age. The disease is characterized by a brief febrile illness, typical vesicular rashes on the palms, soles, or buttocks, and oropharyngeal ulcers, and it can usually resolve spontaneously. In some rare cases, however, patients may also develop neurological complications such as neurogenic pulmonary edema, aseptic meningitis, and acute flaccid paralysis [4]–[6]. As is well known, the most common etiological agents of HFMD are human enterovirus 71 (EV-A71) and coxsackievirus A16 (CVA16), with different ratios [5], [7]–[9]. However, other EVs in the EV-A and EV-B species may co-circulate and account for a sizeable proportion in the pathogen spectrum of HFMD [10]–[14]. Moreover, the clinical manifestations and pathogenesis of HFMD caused by these EV serotypes may have some discrepancies with typical or common HFMD and are sometimes associated with severe cases and death [10], [11], [13], [15], [16].

Since the large-scale outbreaks of HFMD that occurred in Linyi City, China in 2007, HFMD has received considerable attention and was classified as a category C notifiable infectious disease by the Ministry of Health of China, and an HFMD virological surveillance system was set up in 2008 [17], [18]. In the surveillance system, the pathogen detection was focused on EV-A71 and CVA16, and therefore, information on the other EVs, including their geographical distribution and epidemiological profiles, is very limited. In this study, clinical samples from patients with HFMD were investigated in order to identify the circulating EV serotypes in the HFMD outbreaks in Shijiazhuang City, which is the capital city of Hebei province and is one of the biggest cities in China. The results showed that besides EV-A71 (1574 cases) and CVA16 (866 cases), CVA10 (135 cases) and CVB3 (26 cases) were also the main HFMD pathogens and caused outbreaks during 2010–2012. These results indicate that enhanced EV surveillances should be conducted to meet emergency needs, in case of epidemic outbreaks triggered by these EV serotypes in the future. Therefore, virological surveillances to simultaneously detect more EV serotypes besides EV-A71 and CVA16 are necessary.

## Results

### 

#### 1, The epidemiologic characteristics and pathogen spectrum of HFMD in shijiazhuang city during 2010–2012

Large epidemics of HFMD occurred in Shijiazhuang City since 2010, and from January 2010 to July 2012, total 35955 HFMD patients were reported to the National HFMD surveillance system, with 470 severe cases and 31 death cases. 4045 children were recruited in the study, including all severe cases (470) and all death cases (31). Most of the severe (439) and death (29) cases occurred in 2010, and only 19 severe and 2 death cases occurred in 2011, whereas there was no death case in 2012. Among the 4045 children, the age ranged from 1 month to 15 years old, with 83.5% of the patients (3377 children) being less than 3 years old.

**Figure 1:**
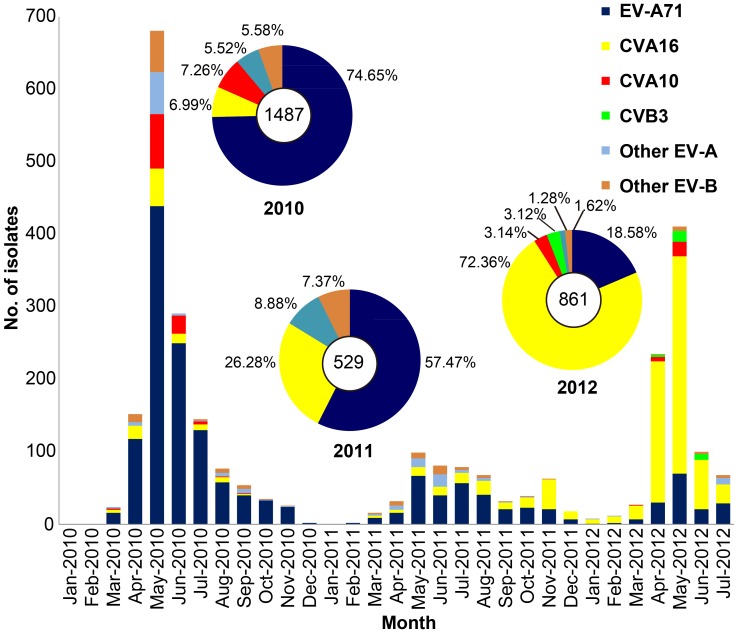
Monthly distribution and constituent ratio of EV serotypes associated with patients with HFMD in Shijiazhuang City, China, during 2010–2012.

Among the samples, 2877 (71.12%) were detected to be positive for EVs, with the most frequently presented serotypes being EV-A71 (1574, 54.71%) and CVA16 (866, 30.10%), and CVA10 (135, 4.69%) was the third and CVB3 (26, 0.90%) the fourth highest in prevalence of the pathogens spectrum that caused HFMD (Figure 1). Co-infection of two viruses (such as EV-A71 and CVA16) at the same time was not found in these samples. Although EV-A71 and CVA16 were the 2 most dominant pathogens that caused HFMD during 2010–2012, the circulating trends of these 2 viruses show a shift in the epidemic features. The spread of EV-A71 became increasingly weak (accounting for 74.65% of all pathogens in 2010 down to 18.58% in 2012), whereas the spread of CVA16 became increasingly stronger (accounting for 6.99% of all pathogens in 2010 up to 72.36% in 2012). There was a positive correlation between the number of HFMD severe and death cases and the proportion of EV-A71 infections detected (*p*<0.05).

#### 2, Sustained transmission of EV-A71 (C4a) and CVA16 (B1a and B1b) in shijiazhuang city

Although the circulating trends of EV-A71 and CVA16 show a shift in Shijiazhuang City during 2010–2012, the transmission of the two viruses never stop, and peaked from April to June every year. The largest outbreaks of EV-A71 and CVA16 occurred in 2010 and 2012, respectively, while the epidemics of the two viruses in 2011 were relatively mild.

**Figure 2:**
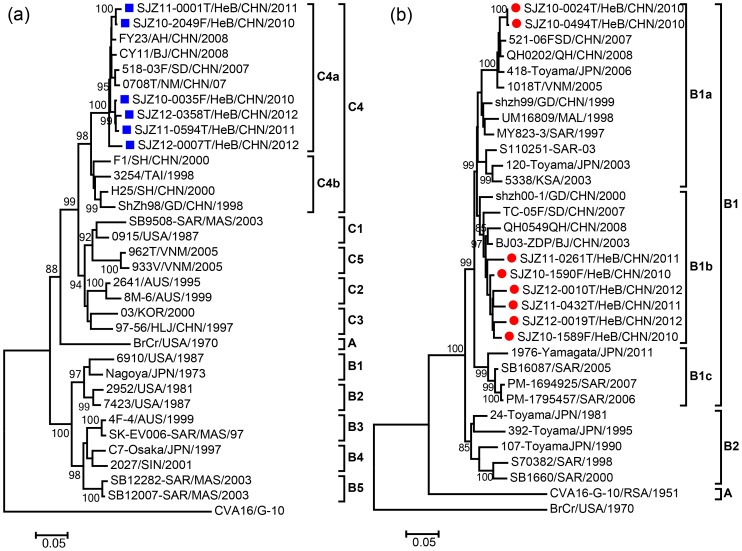
Phylogenetic dendrograms based on entire *VP1* nucleotide sequences (891 bp) of EV-A71 and CVA16. The dendrograms were constructed by using the neighbor-joining method based on the alignment of the entire *VP1* region sequences of Shijiazhuang strains and other strains of known subgenotypes. Bootstrap values (%) for 1000 replicated trees are indicated at the nodes, where only values >80% are shown. (a) Blue square indicate Shijiazhuang HEV71 strains, and the prototype CVA16 strain (G-10) was used as an outgroup. (b) Red circle indicate Shijiazhuang CVA16 strains, and the prototype EV-A71 strain (BrCr) was used as an outgroup.

Phylogenetic analyses were performed based on the complete *VP1* sequences of EV-A71 and CVA16 isolates from Shijiazhuang City and elsewhere, and the analyses showed that the two viruses continued to circulate and evolve over the winter between outbreaks. The molecular epidemiology of EV-A71 and CVA16 in Mainland of China during 2010–2012 reflects the pattern of endemic circulation of subgenotypes C4 (Evolutionary branch C4a) and B1 (Evolutionary branches B1a and B1b) viruses (Figure 2). Evolutionary branch C4a of EV-A71, which is also the predominant type circulating in mainland of China [5], [19], continued to evolve and was responsible for the recent outbreaks with significant morbidity and mortality in 2010 (Figure 2a). All Shijiazhuang CVA16 strains belonged to the co-circulated evolutionary branch B1a and B1b within subgenotype B1 (Figure 2b), which is also the same type circulating in mainland of China [7]. Both EV-A71 and CVA16 strains that circulated in Shijiazhuang City correlated well chronologically with each other and also with EV-A71 and CVA16 strains from other cities in mainland of China, respectively, which suggested that EV-A71 and CVA16 in Shijiazhuang City co-evolved and co-circulated with those from other cities in mainland of China.

#### 3, HFMD outbreaks caused by CVA10 and CVB3 occurred in shijiazhuang city

Besides EV-A71 and CVA16, CVA10 and CVB3were also important pathogens associated with HFMD outbreaks in Shijiazhuang City during the surveillance period. Similar to EV-A71 and CVA16, the prevalent season for CVA10 and CVB3 was the warm season (April to June). CVA10 caused 2 HFMD outbreaks in 2010 and 2012, respectively (Figure 1), with the former outbreak (108 children, laboratory-confirmed) being the bigger one that peaked in May and June 2010. CVB3, on the other hand, caused only a small outbreak (26 children, laboratory-confirmed) from April to June 2012, with the peak incidence occurring in May.

#### 4, Four divergent lineages of CVA10 circulated in shijiazhuang city

Nucleotide sequence alignment of the *VP1* region was performed using 20 Shijiazhuang CVA10 strains isolated from the HFMD patients and CVA10 strains deposited in the GenBank database, including the prototype strain (Strain Kowalik/USA/1950). The pairwise distance among the 20 Shijiazhuang CVA10 strains ranged in divergence from 0.000 to 0.064 and from 0.232 to 0.238, compared with the prototype strain, whereas that of the GenBank strains was from 0.021 to 0.201.

**Figure 3:**
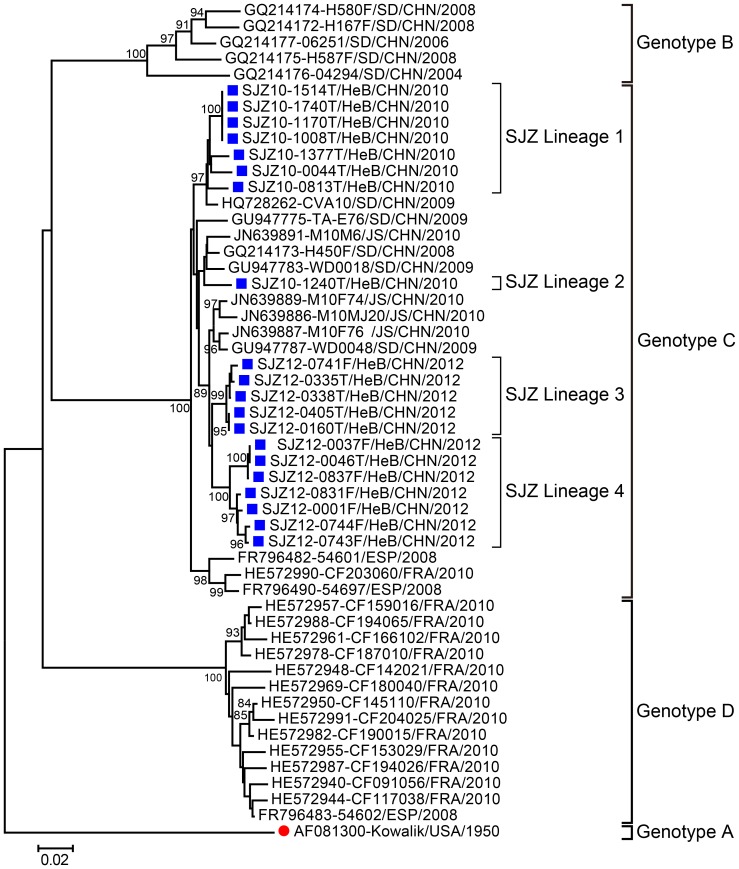
Phylogenetic tree based on the entire *VP1* nucleotide sequences (894 bp) of the CVA10 strains. 20 CVA10 isolates from this study and 32 CVA10 strains worldwide were used to build the tree. Bootstrap values (%) for 1000 replicated trees are indicated at the nodes, where only values >80% are shown. Shijiazhuang isolates are labeled by blue solid squares, whereas the CVA10 prototype strain is labeled by the red solid circle. Abbreviations: SJZ, Shijiazhuang; HeB, Hebei; SD, Shandong; JS, Jiangsu.

Phylogenetic analysis indicated that the CVA10 sequences were assigned to 4 genotypes (A–D), with clear geographical and temporal-specific distributions (Figure 3). All Chinese CVA10 strains were segregated into genotypes B and C: genotype B comprised strains from the Shandong province during 2004–2008; genotype C comprised the strains in this study and other strains from Shandong and Jiangsu provinces during 2008–2012, together with the strains from Spain (2008) and France (2010).

All 20 Shijiazhuang CVA10 strains were derived from the same origin within genotype C and could be divided into 4 lineages. Lineages 1 and 2 contained the strains isolated in 2010, whereas lineages 3 and 4 were made up of the strains isolated in 2012, thus confirming that the CVA10 isolates showed temporal dynamics. This conclusion can be enhanced by the evidence that the strains isolated in 2010 had a 0.034–0.054 genetic divergence with those isolated in 2012. Above all, based on the phylogenetic tree, a high genetic divergence of 4.07±0.06% was found among these 4 lineages, suggesting that at least 4 viral transmission chains of CVA10 had circulated in Shijiazhuang.

#### 5, Shijiazhuang CVB3 has the same origin as those in other provinces of china

The *VP1* region nucleotide sequence alignment was performed using 12 Shijiazhuang CVB3 strains isolated from the HFMD patients and those deposited in the GenBank database, including the prototype strain (Strain Nancy/USA/1949). The pairwise distance among these CVB3 strains ranged from 0.000 to 0.091 and from 0.047 to 0.117, compared with CVB3 strains from other parts of China. Compared with the prototype strain, the genetic divergence ranged from 0.208 to 0.212 and from 0.168 to 0.205, compared with other GenBank CVB3 isolates.

**Figure 4:**
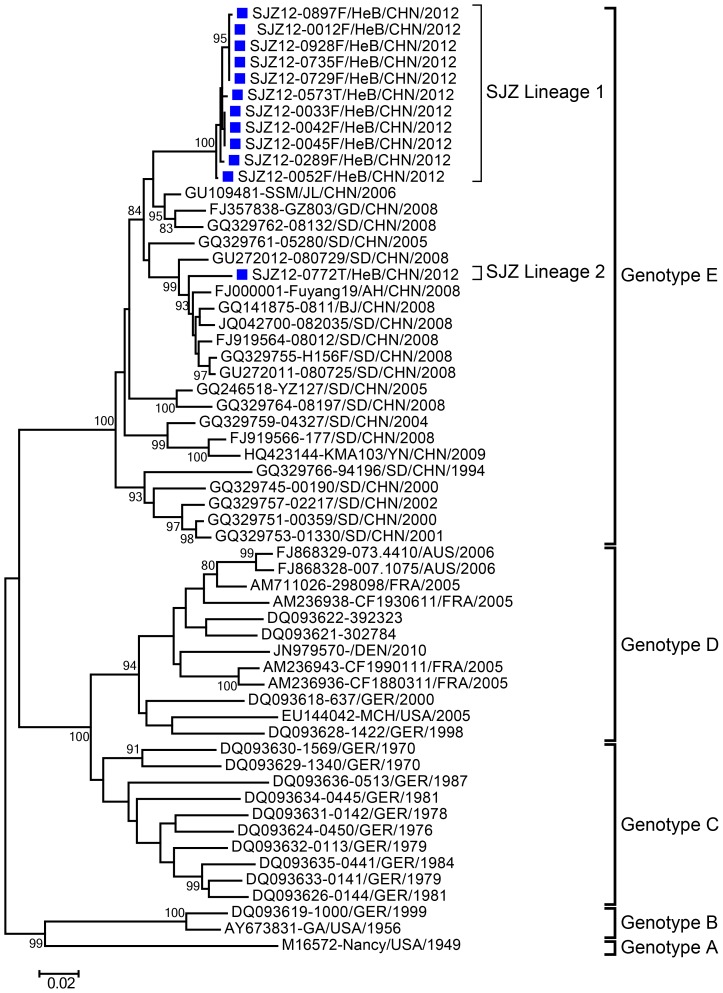
Phylogenetic tree based on the entire *VP1* nucleotide sequences (852 bp) of the CVB3 strains. 12 CVB3 isolates from this study and 46 CVB3 strains worldwide were used to build the tree. Bootstrap values (%) for 1000 replicated trees are indicated at the nodes, where only values >80% are shown. Shijiazhuang isolates are labeled by blue solid squares, and the CVB3 prototype strain is labeled by a red solid circle. Abbreviations: SJZ, Shijiazhuang; HeB, Hebei; JL, Jilin; GD, Guangdong; SD, Shandong; AH, Anhui; BJ, Beijing; YN, Yunnan.

Phylogenetic analysis indicated that the CVB3 sequences were assigned to 5 genotypes (A–E), with clear geographical and temporal-specific distributions (Figure 4). All Chinese CVB3 strains were segregated into genotype E, which comprised the strains in this study as well as other strains from the Jilin, Guangdong, Shandong, Anhui, Beijing, and Yunnan provinces of China during 1994–2012.

There were 2 CVB3 lineages circulating in Shijiazhuang City and the nucleotide divergence among them was 8.8%, which indicate that at least 2 viral transmission chains of CVB3 had circulated. It is very clear that the Shijiazhuang CVB3 strains themselves have the same genetic origin, and have the same genetic origin as the CVB3 strains isolated in other provinces of China such as Shandong, Guangdong, Yunnan, and Anhui provinces. All Chinese CVB3 isolates (including Shijiazhuang CVB3 in this study) clustered as an independent genetic group supported by a high bootstrap value (100%), which was completely separate from the CVB3 strains from other countries such as Germany, France, the USA, and Australia.

## Discussion

Besides EV-A71 and CVA16, other human enteroviruses also account for sporadic cases of HFMD and the occasional outbreak events. CVA10 and CVB3, 2 pathogens that were previously infrequently detected, were identified as pathogens causing the HFMD outbreaks in Shijiazhuang City during 2010–2012. Numerous outbreaks of HFMD have occurred in countries in the west pacific region since 1997, where EV-A71 and CVA16 were the most commonly responsible EV serotypes. EV-A71 specifically was believed to be the pathogen associated with most of the severe cases and almost all of the death cases [4], [20], [21]. However, many studies indicated that, besides EV-A71 and CVA16, several other EVs such as CVA10 and CV-A6 had been frequently detected in specimens collected from patients who presented with HFMD.

In this study, the intensive surveillance for HFMD has permitted a detailed analysis of the outbreaks by molecular epidemiological methods. As a result, CVA10 and CVB3, 2 pathogens that were previously infrequently detected, were identified as pathogens causing the HFMD outbreaks. CVA10 was the third and CVB3 the fourth highest in prevalence of the pathogens spectrum that caused HFMD, which indicated that they were prevalent in Shijiazhuang during 2010–2012.

Our study also identified distinct genotypes of CVA10 and CVB3 that related to their geographical origins. Chinese CVA10 and CVB3 strains formed genotypes different from those in Europe and the USA. The phylogenetic analysis of CVA10 and CVB3 strains that had circulated in Shijiazhuang City showed that they can be clustered into 4 and 2 lineages, respectively. The nucleotide divergences between Shijiazhuang CVA10 and CVB3 and the corresponding virus serotype strains reported in other countries were relatively high. Further evolutionary studies, representing many more geographical locations and different time distributions, would help to improve our understanding of the evolutionary relationships of these strains.

The persistent outbreaks of HFMD have been causing emerging nationwide epidemics since 2007, and HFMD epidemics might persist for a long time in mainland China owing to the multiple pathogen compositions, the enteroviral characteristics of recombination and co-infection, the ever-increasing number of travelers, and the absence of an effective vaccine [11], [15]. Enhanced EV surveillances are warranted to predict the potential of these strains in causing outbreaks, and the composition of the HFMD pathogen spectrum and epidemic pattern of EVs should be further detailed and comprehensively researched. Thus, this study underscores the need for the enhancement of molecular detection for HFMD diagnosis and for detailed laboratory-based surveillances of HFMD in mainland China.

## Materials and Methods

### Viruses

This study did not involve human participants or human experimentation; the only human materials used were throat swab samples and stool samples collected from patients with suspected HFMD, at the instigation of the Ministry of Health P. R. of China for public health purposes. Written informed consent for the use of their clinical samples was obtained from all patients involved in this study. This study was approved by the second session of the Ethics Review Committee of the National Institute for Viral Disease Control and Prevention, Chinese Center for Disease Control and Prevention.

The clinical samples were processed according to standard protocols. The viruses isolates (including EV-A71, CVA16, CVA10 and CVB3) used in this study were isolated from the stool or throat swab specimens collected from the HFMD patients in Shijiazhuang City during 2010–2012. Viruses were isolated from the original specimens by propagation in human rhabdomyosarcoma (RD) and human larynx carcinoma (HEp-2) cells, using conventional methods, and then sequenced.

### Molecular Typing of EV Serotypes in HFMD Specimens

Viral RNA was extracted from the stool specimens or throat swab specimens using a QIAamp Viral RNA Mini Kit (Qiagen, Valencia, CA, USA), and one-step RT-PCRs were performed to detect EV RNAs, using the Access RT-PCR system (Promega, USA). The primer pairs used are listed in Table 1: EV-A71-VP1-S and EV-A71-VP1-S to detect EV-A71 [5], CVA16-VP1-S and CVA16-VP1-A to detect CVA16 [7], E486 and E488 to detect EV-A, and E490 and E492 to detect EV-B [22].

**Table 1:** PCR and sequencing primers used in this study.

Primer	Nucleotide position (nt)	Primer sequence (5′–3′)	Orientation	Reference
EV71-VP1-S	2372–2392	GCAGCCCAAAAGAACTTCAC	Forward	[5]
EV71-VP1-A	3434–3454	AAGTCGCGAGAGCTGTCTTC	Reverse	[5]
CVA16-VP1-S	2335–2354	ATTGGTGCTCCCACTACAGC	Forward	[7]
CVA16-VP1-A	3426–3445	GCTGTCCTCCCACACAAGAT	Reverse	[7]
E486	2297–2322	TGGTAICARACIAAITWYGTIGTNCC	Forward	[22]
E488	3038–3063	GTIGGRTAICCITCITARAACCAYTG	Reverse	[22]
E490	2226–2248	TGIGTIYTITGYRTICCITGGAT	Forward	[22]
E492	2934–2953	GGRTTIGTIGWYTGCCA	Reverse	[22]
CVA10-2407Y	2407–2426	ACTGATGAGGTGACGCAACA	Forward	This study
CVA10-3400Z	3381–3400	CCAGGTGCCTATTGACCACT	Reverse	This study
CVB3-2289S	2289–2308	ACCGCAGGGGGTTTTATTAC	Forward	This study
CVB3-3748Q	3729–3748	ACTCCCTGTTCCATTGCATC	Reverse	This study

### Determination of the Entire *VP1* Nucleotide Sequences of CVA10 and CVB3

Amplicons containing the complete *VP1* region of CVA10 and CVB3 were amplified by conventional RT-PCR methods, using the primer pairs CVA10-2407Y/CVA10-3400Z and CVB3-2289S/CVB3-3748Q, respectively (Table 1). The PCR products were purified using the QIAquick gel extraction kit (Qiagen, USA), and the amplicons were bidirectionally sequenced using an ABI PRISM 3130 genetic analyzer (Applied Biosystems, Hitachi, Japan).

### Phylogenetic Analyses

Multiple entire *VP1* sequences alignments of the viruses (EV-A71, CVA16, CVA10 and CVB3) were constructed using the Molecular Evolutionary Genetics Analysis Version 5.0 (MEGA 5.0) [23]. Phylogenetic trees were constructed by the neighbor-joining method implemented in the MEGA program, using the Kimura 2-parameters model for nucleotide substitution. The branch lengths of the dendrogram were determined from the topologies of the trees and were obtained by majority rule consensus among 1000 bootstrap replicates. Bootstrap values greater than 80% were considered statistically significant for grouping. Scale bar indicates the number of nucleotide substitutions per site.

### Nucleotide Sequence Accession Numbers

The entire *VP1* nucleotide sequences of the 32 Shijiazhuang EV isolates (20 CVA10 and 12 CVB3) that were isolated from the clinical specimens of HFMD patients in this study were deposited in the GenBank database under the accession numbers KF246647 to KF246678.
